# Selection of Nectar Plants for Use in Ecological Engineering to Promote Biological Control of Rice Pests by the Predatory Bug, *Cyrtorhinus lividipennis*, (Heteroptera: Miridae)

**DOI:** 10.1371/journal.pone.0108669

**Published:** 2014-09-25

**Authors:** Pingyang Zhu, Zhongxian Lu, Kongluen Heong, Guihua Chen, Xusong Zheng, Hongxing Xu, Yajun Yang, Helen I. Nicol, Geoff M. Gurr

**Affiliations:** 1 State Key Laboratory Breeding Base for Zhejiang Sustainable Pest and Disease Control, Institute for Plant Protection and Microbiology, Zhejiang Academy of Agricultural Sciences, Hangzhou, China; 2 Jinhua Plant Protection Station, Jinhua, China; 3 Crop and Environmental Sciences Division, International Rice Research Institute, Metro Manila, Philippines; 4 School of Agriculture & Wine Sciences, Charles Sturt University, Orange, New South Wales, Australia; 5 Institute of Applied Ecology, Fujian Agriculture and Forestry University, Fuzhou, Fujian, China; 6 Graham Centre for Agricultural Innovation (New South Wales Department of Primary Industries and Charles Sturt University), Orange, New South Wales, Australia; Swedish University of Agricultural Sciences, Sweden

## Abstract

Ecological engineering for pest management involves the identification of optimal forms of botanical diversity to incorporate into a farming system to suppress pests, by promoting their natural enemies. Whilst this approach has been extensively researched in many temperate crop systems, much less has been done for rice. This paper reports the influence of various plant species on the performance of a key natural enemy of rice planthopper pests, the predatory mirid bug, *Cyrtorhinus lividipennis.* Survival of adult males and females was increased by the presence of flowering *Tagetes erecta*, *Trida procumbens, Emilia sonchifolia* (Compositae), and *Sesamum indicum* (Pedaliaceae) compared with water or nil controls. All flower treatments resulted in increased consumption of brown plant hopper, *Nilaparvata lugens*, and for female *C. lividipennis*, *S. indicum* was the most favorable. A separate study with a wider range of plant species and varying densities of prey eggs showed that *S. indicum* most strongly promoted predation by *C. lividipennis.* Reflecting this, *S. indicum* gave a relatively high rate of prey search and low prey handling time. On this basis, *S. indicum* was selected for more detailed studies to check if its potential incorporation into the farming system would not inadvertently benefit *Cnaphalocrocis medinalis* and *Marasmia patnalis*, serious Lepidoptera pests of rice. Adult longevity and fecundity of both pests was comparable for *S. indicum* and water treatments and significantly lower than the honey solution treatment. Findings indicate that *S. indicum*is well suited for use as an ecological engineering plant in the margins of rice crops. *Sesame indicum* can be a valuable crop as well as providing benefits to *C. lividipennis* whilst denying benefit to key pests.

## Introduction

Ecological engineering for pest management involves experimentation to identify from among a number of agronomically feasible options, the optimal form of diversity for incorporation into a farming system to suppress pests, often by promoting their natural enemies. Whilst this approach has been extensively researched in many temperate crop systems, few studies have been done for rice, despite a mounting need [1], [2]. Rice planthoppers (Hemiptera: Delphacidae) including the brown planthopper (*Nilaparvata lugens* (Stål) ), white-backed planthopper (*Sogatella furcifera* (Horváth)), and small brown planthopper (*Laodelphax striatellus* (Fallén)), are the most destructive insect pests of rice in Asia and outbreaks have occurred frequently in the last decade [3], [4], as a result of insecticide resistance and a break down in resistance genes in rice cultivars [3]. Planthopper impact is so severe that it is now considered to be a substantial threat to world food security [5].

Ecological engineering for pest management has roots in traditional forms of mixed farming systems, but in the context of industrialized agriculture, has been actively pursued under the terms ‘habitat management/manipulation’ and ‘conservation biological control’ for only a few decades [6]. Whilst some forms of diversity manipulation can have a direct suppressive effect on pest populations, most strategies involve conserving natural enemies and improving their performance by providing resources including shelter [7] and, most importantly, the plant derived foods of pollen and nectar [8]. These foods can have a great influence on natural enemy longevity, fecundity and behaviour and lead to an impact on pest numbers [9]–[12].

Three key issues influence the use of food plants in agricultural systems as a means to boost biological control of pests. First, not all plants provide equivalent benefit to natural enemies. Nectar, for example, must have an appropriate profile of sugars and be produced by flowers that are attractive to the predator or parasitoid and that allow physical access to the nectaries [8]. Second, the plant food must not benefit pest species such as moths. This necessitates the identification of ‘selective’ food plants that support feeding only by key natural enemy species [13], [14]. Third, the plants selected should ideally have benefits beyond pest management such as constituting secondary crops or providing complementary ecosystem services [15]. These issues have driven this branch of applied ecology from the often ‘hit and miss’ use of multiple plant species to a more directed, ecological engineering approach in which laboratory screening is often used in order to identify optimal plant species for use in later field experiments [2], [12].

In Asian rice systems, the predatory bug, *Cyrtorhinus lividipennis* (Reuter) (Heteroptera: Miridae) is an important natural enemy of eggs and young nymphs of rice planthoppers [16], [17]. This species is able to survive periods of unavailability of delphacid prey by switching to alternative prey, including conspecifics [18]. Though *C. lividipennis* is generally considered a major predator of delphacid eggs and nymphs, it is also known to attack Lepidoptera eggs and larvae including those of rice leaffolder *Cnaphalocrocis medinalis* (Guene) (Crambidae), rice stem borer *Chilo suppressalis* (Walker) (Crambidae) and pink rice borer *Sesamia inferens* (Walker) (Noctuidae) [19], [20]. Predation rates on the eggs of *N. lugens* by *C. lividipennis* in rice fields can average 30% and reach 70% [21]. Laboratory studies have shown that individual *C. lividipennis* nymphs and adults can consume 7.5 and 10.2 *N. lugens* daily, respectively [22]. Despite the importance of this predator, nothing is known of the life history parameters of *C. lividipennis* that might be improved by access to nectar, the types of food plants that it may benefit from, and whether its impact on pests might be boosted by access to appropriate food plants. Accordingly, the primary aim of this laboratory study was to identify the effects of various plant species on *C. lividipennis.*

Experiments were also undertaken to investigate the extent to which potential nectar plants may provide a benefit to pests. Planthoppers are not able to feed on plants used to border crops; all delphacid pests of rice feed either exclusively on rice or on rice related Poacea. It is Lepidoptera pests that constitute the biggest risk of being inadvertently promoted by the use of inappropriate border plants. *Marasmia patnalis* (Bradley) (Pyralidae) and *C. medinalis* are among the most important Lepidoptera pests of rice in Asia. A wide range of natural enemies attack Lepidoptera pests in rice [23], but provision of adult food resources to these pests could increase crop damage. Nothing is known about the response of the former species to nectar plants but the latter has been shown to feed on some nectar types [24], so there is a potential risk of exacerbating pest problems if inappropriate plant species are selected. Thus, a complementary aim of this study was to determine if food plants could selectively provide a benefit to *C. lividipennis* and not to key pests. These plants could then be grown on the bunds surrounding rice fields to promote biological control of planthopper pests.

## Materials and Methods

### Plants

A pest susceptible variety of rice (TN1) was obtained from the International Rice Research Institute (IRRI), Philippines. After indoor germination, seeds were sown in a cement tank inside an insect proof room. Fourteen-day old rice plants were transplanted into 5 cm diameter clay pots filled with garden soil and grown on to 45-day old rice plants for use in experiments.

Seeds of the crop or medicinal herb species *Tagetes erecta* L, *Trida procumbens* L., *Ageratum conyzoides* L. and *Emilia sonchifolia* (L.) DC. (Compositae) were collected from the IRRI farm located at Los Baños, Philippines (14.4322°N, 120.985°E). Seeds of a landrace of the crop plant sesame (*Sesamum indicum*) were collected from the field at Jinhua, Zhejiang Province, China (29.0833° N, 119.6500° E). The landholders gave permission for the collection of seeds and none of the species are endangered or protected. Commercial seed of *Portulaca grandiflora* Hook (Portulacaceae) was used. All seeds were sown in clay pots (14 cm diameter) filled with garden soil and thinned to two plants per pot at ten days after emergence, then grown in a shaded greenhouse without supplementary lighting, humidity or temperature regulation other than ventilation. Temperature ranged between 25–40°C and relative humidity from 70%–90%. All six plant species (Table 1) were used in studies of insect functional response. A sub-set of four species (*T. erecta*, *T.procumbens*, *E.sonchifolia* and *S. indicum*) was used in other experiments when flowering. *A. conyzoides* and *P. grandiflora* were not available. Later experiments examined the effects of *S. indicum* only on lepidopteran pests on the basis that this plant species appeared most strongly beneficial to *C. lividipennis.* Age of candidate nectar plants at the time of experiments ranged from 40–60 days and height ranged from 15–40 cm. For experiments that used whole plants, each had at least three open flowers present throughout the duration of the experiment.

**Table 1:** Details of plant species tested for utility in ecological engineering against rice pests.

Plant species	Family	Origin^1^	Value
*Tagetes erecta* L.	Compositae	IRRI 14.4322°N,120.985°E	Ornamental/Medicinal herb
*Trida procumbens* L.	Compositae	IRRI 14.4322°N,120.985°E	Medicinal herb
*Ageratum conyzoides* L.	Compositae	IRRI 14.4322°N,120.985°E	Medicinal herb
*Emilia sonchifolia* (L.) DC.	Compositae	IRRI 14.4322°N,120.985°E	Medicinal herb
*Sesamum indicum*L.	Pedaliaceae	Jinhua, China 29.0833°N, 119.650° E	Crop
*Portulaca grandiflora*Hook	Portulacaceae	Proprietary seed	Ornamental

1 IRRI = International Rice Research Institute, Los Baños, Philippines. The landholders gave permission for the collection of seeds and none of the species was endangered or protected.

### Insects

#### Predators

Adult *C. lividipennis* were collected from rice fields of the IRRI farm and cultured on 45–60 day-old plants of susceptible rice (cv. TN1) bearing *N. lugens* eggs. Rice plants with *C. lividipennis* eggs were moved into a breeding cage (50 cm× 38 cm× 80 cm) for hatching, and the offspring reared on the TN1 rice plants with a diet of *N. lugens* eggs. Mirid rearing took place in a greenhouse (average temperature 27.0 ± 5°C, 70–90% RH, 12D: 12L). Predators were sexed ca. 2 hr before use in experiments.

#### Pests

Adults of *N. lugens* were collected from Laguna, Philippines, cultured in a cage with TN1 rice plants as described above. After three days, the plants were moved to another rearing cage and new TN1 seedlings provided regularly to maintain *N. lugens* numbers. Larvae of rice leafroller (*M. patnalis*) were collected from rice fields of the IRRI farm and cultured on caged rice plants grown as described above. Larvae of rice leaffolder (*C. medinalis*) were collected from rice fields at Jinhua, Zhejiang Province, China, and cultured as described for *M. patnalis*. Lepidoptera rearing took place in an insectary (average temperature 26.0 ± 1°C, 70–90%RH, 12D: 12L).

### Effect of flowering plants on *C. lividipennis* adult longevity

*Cyrtorhinus lividipennis* that had emerged up to 6 hr previously were placed individually into cylindrical mylar film cages (12.5 cm high, 7 cm diameter, and with a 1.5 cm diameter access port) that were allocated to six treatments. Flower treatments (*S. indicum*, *T. erecta*, *E. sonchifolia* and *T. procumbens*) used freshly-collected flowers kept turgid by placing the cut end within water-soaked cotton wool. Flowers and water-soaked cotton wool were renewed every 24 hr during the experiment. Preliminary testing established that this water was available to and utilized by insects. A water only treatment had the water-soaked cotton wool but no plant material. A control treatment had neither flower nor water. The access ports of cages were closed with a dry cotton wool swab. Cages were laid out in a fully randomized design in a climate room at 26.0 ± 1°C, 70–90%RH, 12D: 12L. Predator survival was recorded at 2 hr intervals until all individuals died. Mean survival time was then calculated. All treatments had more than forty concurrent replicates.

### Effect of flowering plants on *C. lividipennis* predation of *N. lugens* eggs

Potted 45-day-old rice plants bearing *N. lugens* eggs were prepared by confining five gravid planthoppers to individual rice plants for 24 hr. These insects were then removed and each egg-infested plant was covered with a PVC tube (45 cm high and 14 cm diameter). Five treatments were applied to tubes: flowers of *S. indicum*, *T. erecta*, *E. sonchifolia* and *T. procumbens* plus a control treatment without plant material. A newly emerged male or female *C. lividipennis* was then introduced. All treatments included a water-soaked cotton wool swab. Treatments were replicated 30 times and arranged in a fully randomized design in a shaded greenhouse (average temperature 27.0 ± 5°C, 70–90% RH, 12D: 12L). To give 30 replications, 10 replications were run concurrently, then two additional batches of 10 replications over successive days. After 24 hours of exposure to predators, a microscope (10x) was used to count the number of consumed (flattened, empty chorion) and non-consumed prey eggs within the tissue of the excised plant sheath in the laboratory. The total of these two counts was used to determine the initial number of eggs presented to predators in each experimental unit (cage).

### Effect of flowering plants on *C. lividipennis* functional response

Potted rice plants bearing *N. lugens* eggs were prepared as described above but differing densities of *N. lugens* (1, 2, 4, 6, 8, or 10 adults per cage) were used to give a range of prey egg densities on the rice plants and allowed the functional response to be examined. Seven treatments were applied: flowering shoots of *S. indicum*, *T. erecta*, *E. sonchifolia*, *T. procumbens*, *A. conyzoides* and *P. grandiflora* plus a control treatment without plant material. All treatments included a water-soaked cotton wool swab. Treatments were replicated 15–18 times and arranged in a fully randomized design in the previously described shaded greenhouse (average temperature 27.0 ± 5°C, 70–90% RH, 12D: 12L). A newly emerged male or female *C. lividipennis* was then introduced into each cage. Between four and six replications were conducted at a time and the entire study was completed within 6 days. Numbers of consumed and non-consumed prey eggs were counted as described above after 24 hours of exposure to predators.

### Effect of plants with and without flowers on *C. lividipennis* predation of *N. lugens* nymphs

Four experiments were set up in the same manner as described above for the study of prey egg predation. Each experiment used one food plant species (*S. indicum*, *T. erecta*, *E. sonchifolia* or *T. procumbens*) and had four treatments: (i) a plant with flowers from which nectar was available (as in earlier experiments) (ii) a plant from which inflorescences and buds were removed and (iii) a control with no plant material (iv) predator free control. Thirty newly hatched *N. lugens* nymphs and (except for treatment iv) one *C. lividipennis* adult, were released onto the rice plant in each experimental arena. Numbers of surviving nymphs were recorded after 10 days. Treatment iv provided an estimate of the natural mortality of nymphs over the ten-day period; a parameter that could not be determined from the treatments with a predator because *C. lividipennis* feeds by suctorial removal of some or all of the prey contents leaving behind a cadaver that looks identical to the desiccated cadaver of a nymph that died naturally. A corrected predation mortality value (T_m_) could then be calculated using the formula T_m_ = T_C_-(30-T_CK_), where T_C_ is the final number of live nymphs in treatments with a predator, T_CK_ is the final number of live nymphs in the predator-free treatment and 30 the initial number of prey. All treatments had fifteen concurrent replicates.

### Effect of flowering plants on *C. medinalis* and *M. patnalis* adult longevity and fecundity

A pair of *C. medinalis* or *M. Patnalis* adults that had emerged up to 12 hr previously were placed into cylindrical mylar film cages (12.5 cm high, 7 cm diameter) and allocated to three treatments: a flower of *S. indicum* (renewed each 24 hr) plus water, a 10% V/V honey water solution, and a water only control. Cages were laid out in a fully randomized design with 15–20 concurrent replicates in a climate room at 26.0 ± 1°C, 70–90% RH 12D: 12L. Moth survival was recorded at 6 hr intervals until all died. The total number of eggs laid was also recorded. The nutrient sources were changed daily.

### Data analysis

A survival analysis was used to compare the effect of the food resources on the longevity of *C. lividipennis* by calculating the Kaplan–Meier estimates of the survival function. ANOVA was used to test for treatment effects on *C. lividipennis* longevity and predation of *N. lugens* nymphs and a Tukey post-hoc test used to compare means. Generalised linear mixed models (GLMM) were used to test for differences between the three batches of temporal replicates and between treatments for the initial numbers of eggs presented to predators and the numbers of eggs attacked in the egg predation experiment. The generalised linear component assumed a Poisson distribution with a log link. The random terms were time and replication whilst the fixed term was treatment.

Functional response data were modeled using nonlinear regression. Poisson error variance was assumed. A simplified form of Rogers [25] random predator equation y = A–B*Exp(−K*Eggs) (where A is the predicted asymptote value for maximum daily consumption of prey eggs, A–B is the rate of change for the fitted line and K is its curvature) was used to test for differences between treatments. The analysis first fitted a common line to all the treatments, then fitted a different asymptote (A) for each treatment. It then generalized the model further to apply different rate parameters (B) for each treatment. The final model included different shape parameters (K), so that all the parameters differed between treatments. Changes in residual deviance at each modelling level were tested for significance. For each of the A, B and K parameters, where the analysis showed significance, a planned post-hoc comparison using least significant differences (*P* = 0.05) was made to test for differences between values. Individual curves for each treatment were calculated and plotted.

Survival analysis and ANOVA were performed using SPSS, Statistical v18.0.0. GLMM and fitting the exponential curves was performed using Genstat [26].

## Results

### Effect of flowering plants on *C. lividipennis* adult longevity

Survival curves of *C. lividipennis* differed significantly between treatments for both female and male insects (Females: (log-rank = 380.95, *P<*0.001; Wilcoxon (Breslow) = 342.83, *P<*0.001). Males: (log-rank = 54.84, *P<*0.001; Wilcoxon (Breslow) = 56.55, *P<*0.001). Mean adult longevity of *C. lividipennis* was short for insects in the water only and nil controls, in the order of one to two days, whilst access to flowers of *S. indicum*, *T. procumbens*, *E*. *sonchifolia*, and *T*. *erecta* improved this important life history parameter (Table 2). Mean female longevity of *C. lividipennis* was more than 50 hr in each of the flower treatments, significantly greater compared with water or nil controls (Table 2) (df = 5,351, *F* = 60.426, *P*<0.001). For males, *S. indicum* was the only flower treatment that gave a significantly greater mean (24.8%more) than the water treatment (df = 5,351, *F* = 17.933, *P*<0.001). In the *S. indicum* treatment, *C. lividipennis* males had an average life span of 60 hr compared with less than 48 hr in the water and 17 hr in the nil controls.

**Table 2:** Effects of different flowers on the longevity of *C. lividipennis*.

Treatment	Longevity of female adult (hr)	Longevity of male adult(hr)
*S. indicum*	52.3±2.19 b	60.4±4.37 a
*T. erecta*	60.5±2.94 a	53.4±3.86 ab
*E. sonchifolia*	56.8±2.61 ab	49.9±2.37 b
*T. procumbens*	57.1±2.44 ab	47.3±1.74 b
Water	33.0±2.22 c	48.4±2.54 b
Control	19.4±0.54 d	17.8±0.39 c

Values are mean ± SE. Means within a column followed by differing letters are differ significantly at *P*<0.05. Tukey test was used.

### Effect of flowering plants on *C. lividipennis* predationof *N. lugens* eggs

**Figure 1:**
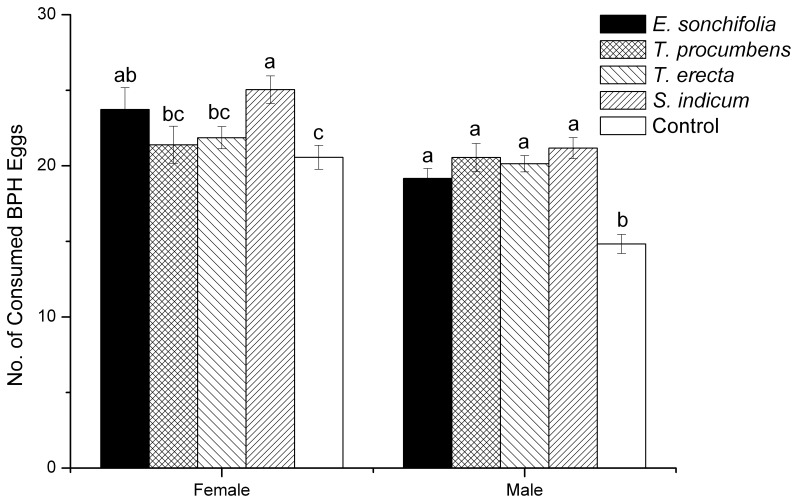
Effect of access to flowering plants of varying plant species on predation by *C. lividipennis*. Adult predators were confined with *ad libitum* brown planthopper eggs plus a flowering shoot or water (control). Numbers of consumed eggs are back transformed.

For female predators, access to flowering shoots of *S. indicum* and *E*. *sonchifolia* gave a significant (df = 4,139, *F* = 3.10, *P* = 0.018) increase in prey consumption compared with the control treatment (Figure 1). For male predators, all four plant treatments significantly (df = 4,100, *F* = 13.09, *P*<0.001) increased the numbers of prey eggs consumed (Figure 1).

The initial numbers of eggs available to female predators in the plant treatments differed significantly (df = 4,139, *F* = 3.368, *P*<0.001) but no plant treatment differed significantly from the control value (Table 3). Accordingly, differences in predation rate between the control and *S. indicum* and *E*. *sonchifolia* treatments are attributable to the imposed treatments and not caused by the initial egg numbers. For male predators the control treatment had the lowest mean initial egg number and differed significantly (df = 4,100, *F* = 3.59, *P* = 0.009) from that in the *S. indicum* treatment (Table 3). Accordingly, any effect of initial egg density on consumption would have led to higher consumption in the control treatment rather than the observed result that consumption was significantly higher in the *S. indicum* treatment.

**Table 3:** Initial numbers of prey eggs available to *C. lividipennis* in a study of the effect of access to flowering plants of varying plant species on predation.

Treatment	Female	Male
*S. indicum*	146.11±7.76 b	129.46±6.34 b
*T. erecta*	151.74±5.45 b	152.57±6.81 ab
*E. sonchifolia*	171.80±6.24a b	140.08±9.24 ab
*T. procumbens*	180.43±9.20 a	161.36±7.24 a
Control	167.15±8.39 ab	160.96±6.50 a

Values are mean ± SE. Means within a column followed by differing letters are differ significantly at *P*<0.05. Tukey test was used.

Temporal runs did not differ in terms of the numbers of eggs consumed by males (df = 2,100, *F* = 0.10, *P* = 0.903; females: df = 2,139, *F* = 0.80, *P* = 0.452) but timing was significant for females (df = 2,139, *F* = 6.66, *P* = 0.002). There was no significant interaction between timing and plant species (df = 8,139, *F* = 1.79, *P* = 0.085).

### Effect of flowering plants on *C. lividipennis* functional response

**Figure 2:**
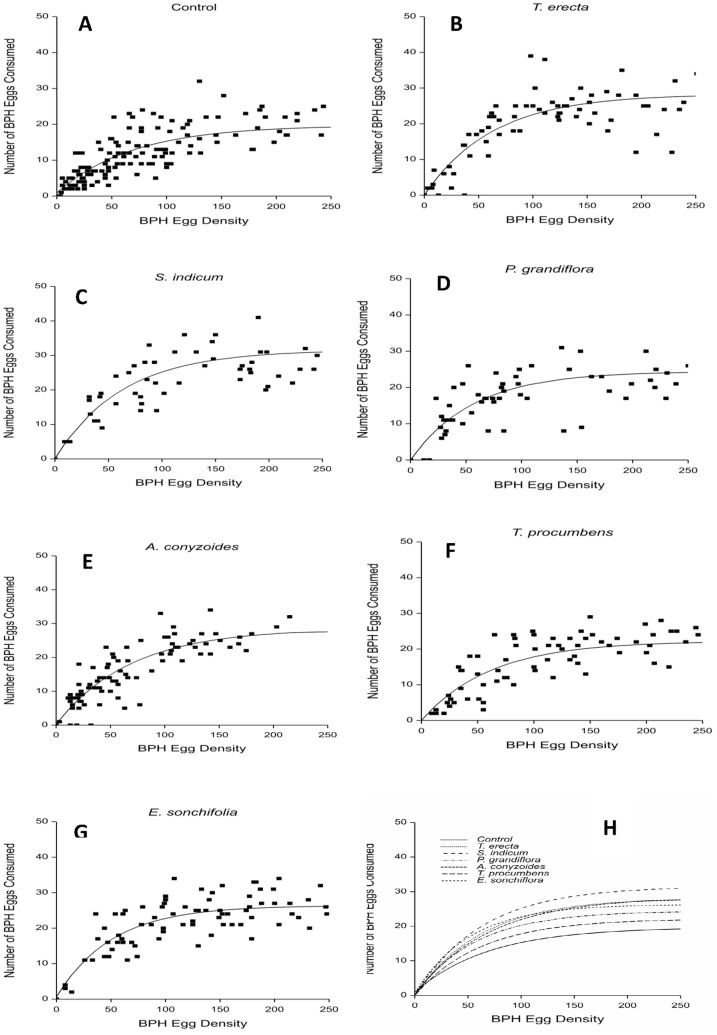
Effect of access to flowering plants of varying plant species on functional response of *C. lividipennis* females. Adult predators were confined with rice plants bearing different densities of brown planthopper (BPH) eggs and numbers of eggs remaining recorded after 24 hr. A: water (control); B: *T. erecta*; C: *S. indicum*; D: *P. grandiflora*; E: *A. conyzoides*; F: *T. procumbens*; G: *E. sonchifolia*; H: comparison of fitted curves for all treatments.

**Figure 3:**
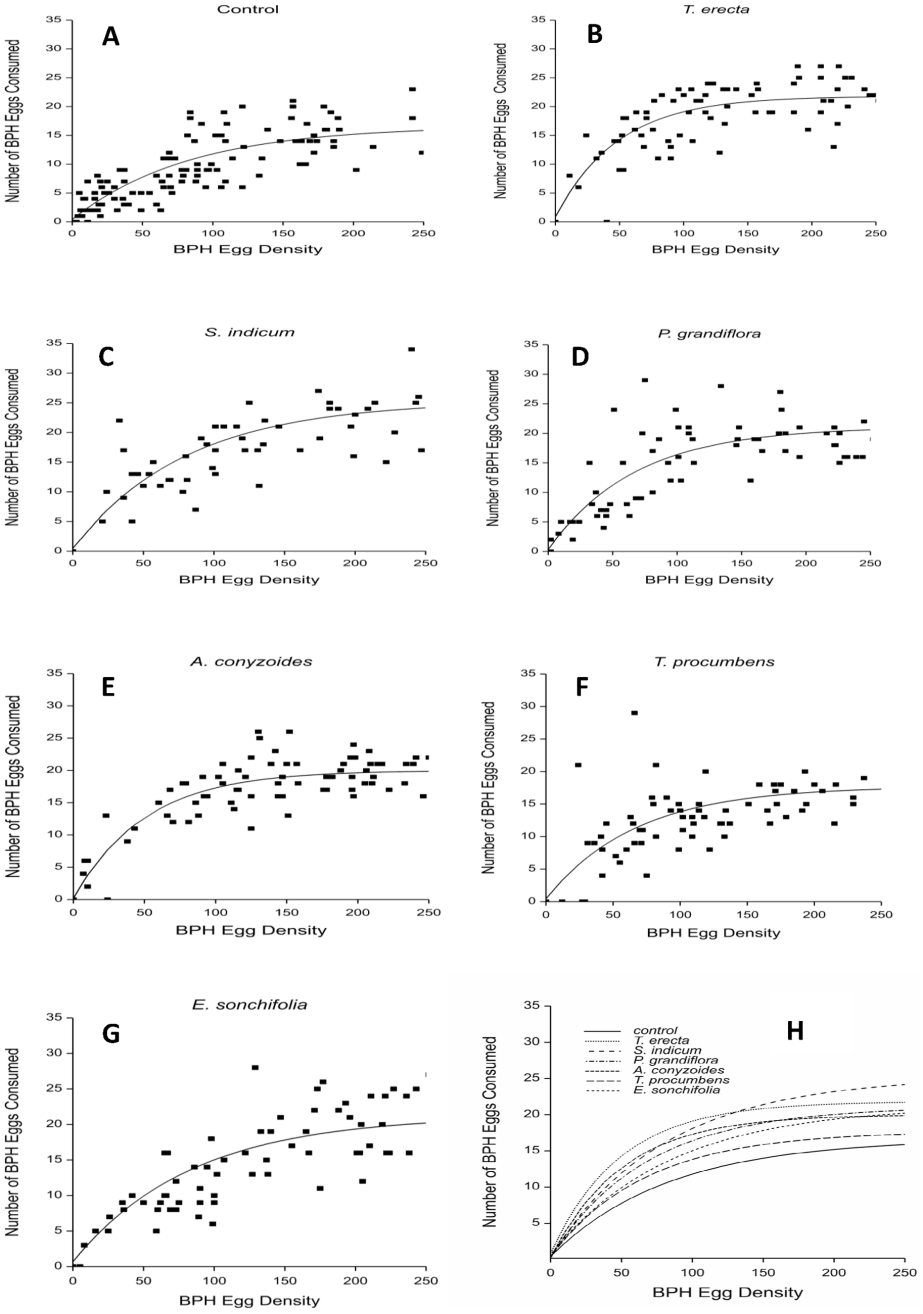
Effect of access to flowering plants of varying plant species on functional response of *C. lividipennis* males. Adult predators were confined with rice plants bearing different densities of brown planthopper (BPH) eggs and numbers of eggs remaining recorded after 24 hr. A: water (control); B: *T. erecta*; C: *S. indicum*; D: *P. grandiflora*; E: *A. conyzoides*; F: *T. procumbens*; G: *E. sonchifolia*; H: comparison of fitted curves for all treatments.

In all diet treatments, the numbers of *N. lugens* eggs consumed by female and male predators increased with prey egg density with data corresponding with Holling’s disc equation II (figures 2 and 3, respectively and tables 4and 5, respectively). The effect of timing (from the fact that not all replicates were concurrent) on numbers of consumed and total number of prey eggs was not significant for any treatment.

Comparison of the curves fitted to each treatment differed significantly for asymptote (maximum daily prey consumption), intercept and curvature for females (df = 20,629, *F* = 86.30, *P*<0.001) and between treatments for asymptote and intercept but not curvature for males (df = 14,654, *F* = 95.34, *P*<0.001). Significant differences in the asymptotes showed females consumed the greatest number of eggs in the *S. indicum* treatment and significantly fewer in the control (df = 6,629, *F* = 30.05, *P*<0.001) (Table 4), and males consumed the greatest number of eggs in the *S. indicum* and significantly fewer in the control (df = 6,649, *F* = 31.56, *P*<0.001) (Table 5).

**Table 4:** Parameter estimates of the functional response of *C. lividipennis* female adult (A–B*EXP(-K*Eggs)).

Treatment	K	B	A (Maximum consumption^1^ (per day))
Control	0.0145 a	18.83 a	19.73 a
*S. indicum*	0.0160 a	31.48 b	31.57 c
*T. erecta*	0.0159 b	28.29 b	28.29 b
*P. grandiflora*	0.0177 b	24.43 b	24.44 ab
*E. sonchifolia*	0.0196 b	25.74 ab	26.36 a
*T. procumbens*	0.0162 a	22.21 ab	22.21 b
*A. conyzoides*	0.0200 a	28.31 c	28.52 c

1 Predicted asymptote value for fitted lines showing maximum numbers of prey eggs consumed.

Values within a column followed by different letters differ significantly based on planned post hoc comparisons (*P* = 0.05 LSDs).

**Table 5:** Parameter estimates of the functional response of *C. lividipennis* male adult (A–B*EXP(-K*Eggs)).

Treatment	K	B	A (Maximum consumption1 (per day))
Control	0.0121 a	16.24 a	16.64 a
*S. indicum*	0.0124 a	24.76 c	25.27 c
*T. erecta*	0.0204 a	20.94 c	21.81 b
*P. grandiflora*	0.0149 a	20.78 b	21.09 a
*E. sonchifolia*	0.0120 a	20.58 bc	21.24 bc
*T. procumbens*	0.0149 a	17.26 b	17.71 a
*A. conyzoides*	0.0199 a	19.83 b	20.01 b

1 Predicted asymptote value for fitted lines showing maximum numbers of prey eggs consumed.

Values within a column followed by different letters differ significantly based on planned post hoc comparisons (*P* = 0.05 LSDs).

### Effect of plants with and without flowers on *C. lividipennis* predation of *N. lugens* nymphs

**Figure 4:**
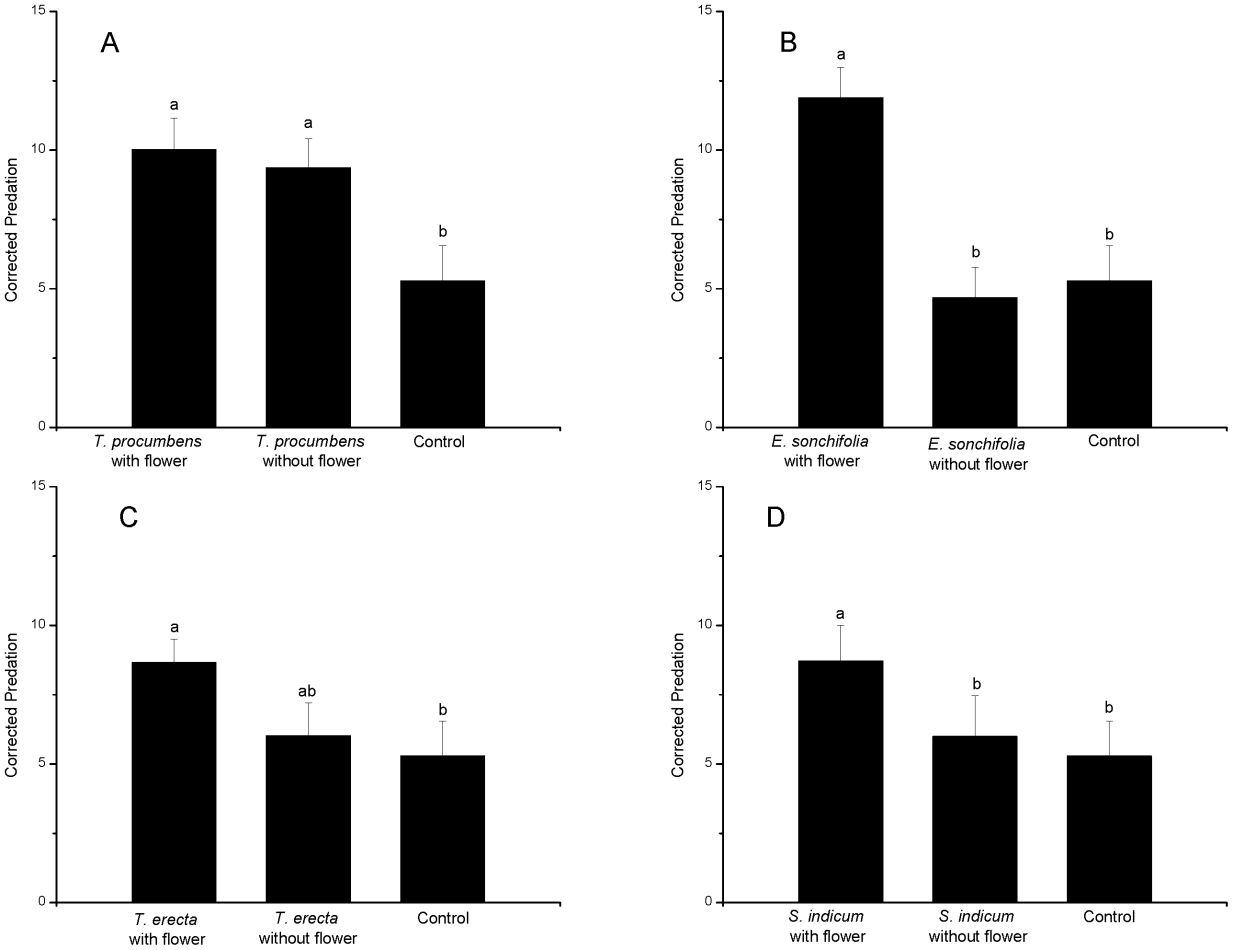
Effect of removing flowers from plants on predation by *C. lividipennis*. Adult predators were confined with brown planthopper nymphs plus either a flowering plant, a plant from which flowers and flower buds were removed or no plant material and mortality assessed after 10 days. A fourth treatment in each experiment had no predator so provided an estimate of background nymph mortality allowing a corrected mortality to be calculated for the other three treatments (see text for explanation). A*: T. procumbens*; B: *E. sonchifolia*; C: *T. erecta*; D: *S. indicum*.

For *S. indicum*and *E. sonchifolia*, predator performance as measured by corrected predation values was significantly improved by the presence of the nectar source compared to the nectar-free plant treatment (df = 2,44, *F* = 3.648, *P* = 0.035; df = 2,44, *F* = 12.088, *P*<0.001, respectively) (Figure 4). In the case of *T. erecta*, corrected predation was intermediate between the nectar and control treatments suggesting that the insects were deriving benefit from the non-flower plant tissues. This conclusion was also supported by the *T. procumbens* results as corrected predation in the flower-free treatment was significantly superior to that in the control and no different to the treatment with nectar (df = 2,44, *F* = 2.590, *P* = 0.012) (Figure 4).

### Effect of flowering plants on *C. medinalis* and *M. patnalis* adult longevity and fecundity

The availability of *S. indicum* flowers to *C. medinalis* and *M. patnalis* adults did not significantly increase their longevity or fecundity compared to the water treatment. Both longevity and fecundity of these pests was significantly higher when provided with a honey solution(longevity: *M. patnalis* female, df = 2,81, *F* = 29.221, *P*<0.001; male, df = 2,81, *F* = 142.539, *P*<0.001; *C. medinalis* female, df = 2,65, *F* = 15.206, *P*<0.001; male, df = 2,65, *F* = 13.865, *P*<0.001; fecundity: *M. patnalis*, df = 2,81, *F* = 33.823, *P*<0.001; *C. medinalis*, df = 2,87, *F* = 14.340, *P*<0.001) (Table 6).

**Table 6:** Adult longevity and fecundity of rice leaffolder, *C.medinalis* and *M. patnalis,* fed on different foods.

Treatment	Adult longevity (hr)				Fecundity (eggs/female)	
	*C. medinalis*		*M. patnalis*		*C. medinalis*	*M. patnalis*
	Female	Male	Female	Male		
10% honey solution	221.1±14.91 a	212.7±17.17 a	246.7±27.97 a	314.1±17.62 a	63.6±13.56 a	64.2±13.04 a
Sesame flower	140.3±10.47 b	117.0±8.97 b	126.2±7.15 b	112.3±5.36 b	0.8±0.43 b	4.6±2.89 b
Water	137.4±45.23 b	107.7±7.78 b	132.3±7.93 b	105.3±4.93 b	0.1±0.10 b	1.1±1.05 b

Values are mean ± SE. Means within a column followed by differing letters are differ significantly at *P*<0.05. Tukey test was used.

## Discussion

The findings of this study illustrate the effects that plant foods can have on natural enemies. *Cyrtorhinus lividipennis* adult longevity and survival pattern, numbers of prey consumed and attack rate on prey were all impacted in a manner consistent with greater levels of biological pest suppression. The present result constitutes a significant contribution to the body of knowledge on omnivorous natural enemies as well as being the first such study of *C. lividipennis.* A previous review of the effect of plant foods on omnivorous natural enemies encompassing 26 studies [27] did not feature this predator species. Indeed only two of the studies reported by [27] were on Miridae, the family to which *C. lividipennis* belongs. Neither of those studies [28], [29] examined the effects of nectar on the predator and neither examined effects on prey consumption. Five more recent studies have investigated the response of predatory Heteroptera to potential food resources. Amongst those concerned with non-mirid species, one study focused on the effects of artificial diets on *Geocoris varius* (Uhler) (Hemiptera: Geocoridae) [30], whilst a second examined the response of *Orius majuscules* (Reuter) (Heteroptera: Anthocoridae) to plant-provided foods [31]. In that study, access to the nectar of sweet alyssum (*Lobularia maritima* L.) alone increased survival of *O. majuscules* compared with a diet of green bean pods and prey eggs (*Ephestia kuehniella* Zeller (Lepidoptera: Pyralididae)) illustrating the potential for appropriate nectar sources to sustain hemipteran predators during times of prey scarcity. Of three recent studies of mirid predator response to diet, one study [32] reported that the addition of sucrose (0.5 M) to a diet of *E. kuehniella* eggs significantly increased *Nesidiocoris tenuis* Reuter progeny but did not affect survival of nymphs and developmental time. Moreover, the addition of sucrose significantly reduced the number of prey eggs consumed. A field experiment on *Macrolophus pygmaeus* Rambur (Hemiptera: Miridae) [33] found that whilst supplementary prey (*E. kuehniella* eggs) enhanced population development, a treatment of fructose plus pollen of *Typha latifolia* L. had no effect. The most recent mirid study, which also focused on *M. pygmaeus* [34], was significant in addressing the capacity of mirids to access plant carbohydrates by direct feeding on plant tissue and thereby giving these natural enemies a wider choice of plant-derived foods than is accessible to other taxa, such as parasitoids, that depend on nectar. That study established that survival of *M. pygmaeus* is extended by access to broad bean plants with extrafloral nectaries compared with broad bean plants without extrafloral nectaries. Notably, the availability of nectar gave survival equivalent to a diet of broad bean plus prey (*E. kuehniella* eggs) as well as increasing predator reproduction. This final study illustrates the potential value of nectar sources to mirid natural enemies, even in the context of there being other types of plant tissue available upon which feeding might occur. More generally, the available information on the response of hemipteran natural enemies suggests that artificial food sources based on sugars or pollen are inferior. Collectively, these findings illustrate that ecological engineering attempts to enhance biological control by predators such as *C. lividipennis* require the identification of appropriate nectar plants.

The honeydew excreted by rice delphacid pests, which is likely the most prevalent source of exogenous sugars available to *C. lividipennis*, cannot be neglected. However, only two publications have investigated the effects of honeydew on *C. lividipennis.* A Japanese study [35] showed that honeydew has a positive effect on the development and reproduction of *C. lividipennis* but did not include other non-prey resources such as nectar so does not allow a comparison of the relative benefits of these two carbohydrate sources. A second study [36] reported that honeydew attracts mirid bugs but does not extend survival. Longevity of *C. lividipennis* was significantly greater on a diet of honey or sucrose (> 8 days) than on honeydew (ca. 5 days). The effect of honeydew on reproduction of *C. lividipennis* has not been examined. More generally, although honeydew might be readily available in rice crops infested with delphacids, this substrate is often nutritionally inferior to nectar as a consequence of a sugar profile that is sub-optimal for natural enemies [37] or the presence of plant allelochemicals [38].

A key issue for the success of predators in biological control of pests is the significance of plant feeding in their ability to suppress prey [27]. If a predator is able to utilize a range of prey types and plant foods, community ecology theory predicts that the predator would be better able to regulate the focal prey species as a consequence of its ability to persist in a given location when the lack of preferred prey would otherwise drive it to extinction or migration [39]. This has two implications. First, its capacity to persist in a given area avoids there being a lag between prey arrival and the onset of predation. Second, its ongoing presence means that the focal pest (planthoppers in the case of *C. lividipennis* and rice) is under constant predation pressure, so preventing it from resurgence. Thus, evidence that *C. lividipennis* utilizes nectar from several species of plant, a phenomenon not previously reported, is of fundamental importance in harnessing the predatory capacity of this insect for biological control of rice pests.

Results from the nymph predation study suggest that, at least under non-choice laboratory conditions, *T. procumbens* can still benefit *C. lividipennis* after flowers and flower buds are removed. This phenomenon is most likely due to the predators piercing the plant and feeding on xylem and mesophyll tissues [34]. There is an abundance of studies showing the positive effects on insect predators, including various Hemiptera, of access to non-floral plant tissues such as leaves, pods, seeds, and the ‘tips’ and ‘squares’ of cotton plants [27]. Importantly, however, access to *S. indicum* and *E.sonchifoilia* flowers gave significantly enhanced insect performance compared to the flower-free treatments of these plant species. This illustrates the additional value of floral food over any nutritional benefit of the vegetative plant material. Future studies will need to determine whether *C. lividipennis* utilises non-nectar plant foods in the field and the practical significance of any such finding.

A theoretical challenge to the notion of the predator boosting its impact on the prey population by nectar feeding is the possibility that plant food may be preferred. This could lead to a net decrease in prey consumption [40]. Such an effect has been demonstrated empirically in a study of the ladybird beetle *Coleomegilla maculata* in corn plots with and without pollen available as a plant food to complement the insect prey *Helicoverpa zea* [41]. When corn plants were de-tasseled so that the predator had access only to insect prey, predation was greater than in plots where pollen was available as a secondary food. Such a negative effect of plant food on predation does not, however, always apply. For example, other work with the same crop and insects showed an increase in predation when the weed *Acalypha ostryaefolia* was present in corn plots [42]. This background points to a potential risk of making plant foods available to *C. lividipennis*: a net reduction in pest suppression could result. In fact, empirical evidence from the present study demonstrates clearly that the simultaneous availability of prey and the flowers of any one of several plant species had the effect of significantly increasing prey consumption.

A large number of studies have illustrated that parasitoid wasps (Hymenoptera) often benefit from access to plant nectar but that the nature and magnitude of benefit can differ markedly with plant species [43]. The present results are the first to show the same phenomenon for a mirid natural enemy though some work has been done on the omnivorous anthocorid bug, *Orius insidiosus* [31]. In that study, insect fecundity, survival, and nutritional status were measured in response to a range of flowers (alyssum, buckwheat, phacelia, fava bean, and chamomile) but prey consumption was not considered.

In the present study, mean longevity of *C. lividipennis* was improved 2–3 fold by all flower treatments compared with the *ca.* one day mean life span in the water control treatment. The absolute levels of predator longevity, as well as other aspects of its performance, need to be interpreted in the context of the artificial setting of the testing conditions. The present study of the effects of nectar on longevity did not provide predators with prey insects as was done in earlier work (which did not examine the effects of plant foods) in which females lived for up to 21 days and males 25 days [22]. Thus the absolute levels of longevity are not comparable across studies. Here we show statistically significant differences in performance as a result of access to flower nectar. Clearly further work is required to establish the magnitude of effects under field conditions.

In contrast to females, in which longevity was increased by all flower treatments, males benefitted significantly only from *S. indicum.* Whilst the performance of males is very much less important that than of females in the case of parasitoid wasps (where only females parasitize hosts), the males of predatory natural enemies are no less important than are females. Because of this, the identification of *S. indicum* as the only plant species among those tested that benefited males is of great significance and reasons for the phenomenon need to be explored in future work. Generally, female insects are more heavily reliant on protein than males because of the need to mature eggs. The dietary protein needs of male insects for sperm production are lower, and often carbohydrate substrates are preferred in order to maximize energy supply for mating-related behaviour [44]. This generalization may help to explain the present results if *S. indicum* had a nectar sugar composition that was particularly attractive and beneficial to male *C. lividipennis* but was no more attractive or beneficial to female insects than the nectar of the other plant species tested. *Sesamum indicum* also gave the greatest effect on the numbers of prey eggs consumed, an observation that applied over a wide range of prey densities.

Food plants established along side rice crops need to bloom as early as possible in the cropping season and continue to produce inflorescences for an extended period. Fortunately in this regard, *S. indicum* blooms in an indeterminate manner, producing new inflorescences higher on the plant as the spike extends over the course of the growing season. Flowering can last at least 60 days (Pingyang Zhu, personal observation) and flower availability could be further extended by sequential sowing. The status of *S. indicum* as the species of choice for promoting the performance of *C. lividipennis* is consistent with recent studies of the potential food plants to enhance *Anagrus* spp. parasitoids of rice planthoppers [45]. In that study, the longevity, realised parasitism and handling time of two parasitoids were enhanced more strongly by *S. indicum* than by other plant species. Reasons for the particularly strong beneficial effects of *S. indicum* are yet to be studied but based on the findings of Begum [46] flower color is possibly important. That study compared the benefits to the parasitoid *Trichogramma carverae* (Oatman & Pinto) of cultivars with differing colors of alyssum (*Lobularia maritime* L). White flowers most strongly supported parasitoid performance, an effect that was diminished when the white petals were colored by placing plant roots in food dye. Of the plants compared in the present study, only *S. indicum* had white flowers (though *T. procumbens* flowers are partly white).

Though food plants can strongly enhance the laboratory performance of a natural enemy, and a flowering crop can affect natural enemy distribution in an adjacent, non-flowering crop [47], these effects do not always lead to pest suppression in the field. Early attempts to promote parasitism of the potato moth (*Phthorimaea operculella* Zeller) with nectar plants exacerbated pest damage to the crop because adult moths were able to take nectar from the border plants and this increased their longevity and fecundity [13]. That finding led to the concept of ‘selective’ food plants that benefit natural enemies but deny benefit to key pests [13], [48]. Lepidoptera pests constitute the biggest risk of being inadvertently promoted by the use of inappropriate border plants. The rice leaffolders are among the most important Lepidoptera pests of rice in Asia. A wide range of natural enemies attack Lepidoptera pests in rice [49], but any factor that provided adult food resources to these pests could dramatically increase crop damage. For this reason, the studies of the effect of flowering *S. indicum* plants on these two pests are of great practical significance. Though the honey solution almost doubled longevity in the present study, and fecundity was raised by more than a hundred-fold compared to a water diet, access to flowering shoots of *S. indicum* did not promote either life history parameter. Thus, this plant species appears safe to use as a ‘selective’ food plant that will deny benefit to key Lepidoptera pests whilst strongly promoting the performance of natural enemies including *C. lividipennis.* The mechanism for this selectivity is not known but work in other flower/Lepidoptera systems indicates that the internal architecture of flowers, nectar composition and the time of the day during which nectar is produced can be important in allowing a directed approach to identifying the appropriate forms of diversity for introduction to a farming system [8], [50], [51].

A further factor supporting the choice of *S. indicum* is that it is agronomically compatible with rice in many parts of Asia and considered a profitable crop; the seeds being used in high value confectionary, beverage and cereal products. This means that its use on the bunds around rice fields to provide nectar to natural enemies would have the additional benefit of providing farmers with a secondary crop. Labor for hand sowing and harvesting is not a major constraint in many Asian countries, so *S. indicum* cultivation in the long narrow confines of bunds around flooded rice is practicable, indeed other crops such as soybean are commonly grown on bunds in some areas [52].

## Conclusion

In China, the world’s largest rice producer, a move towards ‘eco-agriculture’ emphasizes the rational use of inputs [53] and ecological engineering for rice pests has become popular there as well as more widely in east Asia [48]. The present laboratory findings will inform the future selection of appropriate nectar plant species for use in rice systems, particularly since sesame is likely to promote *Anagrus* spp. parasitoids [45], [49] in addition to *C. lividipennis*. The practice of growing crop plants in conjunction with rice exemplifies the enhancement of ecosystem services such as biological control by relatively simple cultural practices [54].
